# A Metagenomic Survey of Viral Abundance and Diversity in Mosquitoes from Hubei Province

**DOI:** 10.1371/journal.pone.0129845

**Published:** 2015-06-01

**Authors:** Chenyan Shi, Yi Liu, Xiaomin Hu, Jinfeng Xiong, Bo Zhang, Zhiming Yuan

**Affiliations:** 1 Key Laboratory of Agricultural and Environmental Microbiology, Wuhan Institute of Virology, Chinese Academy of Sciences, Wuhan, China; 2 University of Chinese Academy of Sciences, Beijing, China; 3 Hubei Disease Control and Prevention Center, Wuhan, China; The Pirbright Institute, UNITED KINGDOM

## Abstract

Mosquitoes as one of the most common but important vectors have the potential to transmit or acquire a lot of viruses through biting, however viral flora in mosquitoes and its impact on mosquito-borne disease transmission has not been well investigated and evaluated. In this study, the metagenomic techniquehas been successfully employed in analyzing the abundance and diversity of viral community in three mosquito samples from Hubei, China. Among 92,304 reads produced through a run with 454 GS FLX system, 39% have high similarities with viral sequences belonging to identified bacterial, fungal, animal, plant and insect viruses, and 0.02% were classed into unidentified viral sequences, demonstrating high abundance and diversity of viruses in mosquitoes. Furthermore, two novel viruses in subfamily Densovirinae and family Dicistroviridae were identified, and six *torque tenosus virus1* in family Anelloviridae, three porcine parvoviruses in subfamily Parvovirinae and a *Culex tritaeniorhynchus* rhabdovirus in Family Rhabdoviridae were preliminarily characterized. The viral metagenomic analysis offered us a deep insight into the viral population of mosquito which played an important role in viral initiative or passive transmission and evolution during the process.

## Introduction

Mosquitoes are important insect vectors to transmit amount of known pathogenic viruses in Family Flavivirus and Alphavirus, such as Dengue virus (DENV), West Nile virus (WNV) and Chikungunya virus (CHIKV), which threaten the public health and gain considerable attention worldwide [1, 2]. During the extrinsic incubation period of transmission cycle, mosquitoes are infected through ingestion of viremic blood from vertebrate host, then the viruses replicate and deposit into apical cavities of acinar cells, inoculating viruses into a new host upon refeeding [3]. In the infection process, viruses are subject to many bottlenecks such as evasion of the host and mosquito immune system which produce a certain evolutionary pressure on viruses leading to their mutation [3]. Since the hosts that mosquitoes feed on are highly diverse including human, livestock, mammals, birds and plants [4], mosquitoes have access to amount of viruses or even novel viruses and are likely to be a viral reservoir or intermediate. In addition, some viruses can also be mechanically transmitted from one host to another by contamination mouthparts or body surface of mosquitoes without infection or replication [3]. So surveillance for viral population in mosquitoes is critical for effective prediction of future vector-borne virus epidemic [5].

Hitherto, viral molecular detection of mosquitoes mainly focuses on greatly harmful and well-studied viruses by using degenerate PCR and real-time PCR [6, 7] which do not require *in vitro* replication and scarce serological or antigenic reagents [8]. These methods are efficient and specific, and can be widely used for identified mosquito-borne viral surveillance. However, the abundance and diversity of viruses as well as their effects on virus evolution and recombination have not been well investigated, and the risks of the identified and/or unidentified viruses on vector-born epidemic have not been evaluated. In consideration of the complex living environment of mosquitoes and their great virus carrying potential, it is significant to explore how abundant and diverse of viruses in mosquitoes.

With the development of second generation DNA sequencing technology, viral metagenomic analysis overcoming the limitation of traditional methods in viruses identification is considered to be an effective method not only for studying viral composition in various samples, such as respiratory tract [9, 10], feces [11, 12], blood [13], animal tissue [14], plants [15], *etc* or a specific biotope [16] but also for discovering novel viruses [17]. Previous researches demonstrated the biologically present of numerous viruses in a specific sample, and the cross transmission of viruses from one species to another which may initiate viral infectious diseases [18]. However, application of this technology in the characterization of viral flora in mosquito is rare yet. In previous study, viral metagenomic analysis was performed on mosquito samples collected in San Diego, USA to explore DNA virus population in them, reflecting a broad range of animal, plant, insect and bacterial viruses [5]. Besides, a novel densovirus infecting *Culex erythrothorax* was identified and animal viruses including anelloviruses, circoviruses, herpesviruses, poxviruses and papillomaviruses were detected using this technique.

In this study, the abundance and diversity of viruses in three mosquito samples from Hubei, China, were analyzed by metagenomic sequencing.The novel mosquito densovirus and Big Sioux River virus were identified, and six *torque teno sus virus 1*, three porcine parvoviruses and a *Culex tritaeniorhynchus* rhabdovirus were characterized. The data provides a preliminary baseline of DNA and RNA virus community in mosquitoes, as well as the animal and plant host they feed on. Highly diversity of viral flora in mosquitoes, as a widespread vector, underlines its function in virus transmission and evolution.

## Results and Discussion

### Overview

1,202 mosquitoes were collected at four locations in Hubei province during July 2013. Sample I was a collection of *Culex tritaeniorhynchus*. Sample II was *Anopheles sinensis*. Sample III was a mixture pool of *Armigeres subalbatus* and *Culex fatigans* (Table 1). The four species are the principal vectors of Japanese encephalitis virus (JEV) and *Plasmodium vivax* in Hubei.

**Table 1:** Mosquito Samples used for metagenomic analysis.

Samples	Species	Numbers	Locations
Sample I	*Culex tritaeniorhynchus*	438	Hubei province^a^
sample II	*Anopheles sinensis*	424	Hubei province^a^
Sample III	*Armigeressubalbatus*	259	Hubei province^a^
	*Culex fatigans*	92	Hubei province^a^

^a^Collection sites include Zhouji (N 30° 24’, E 112° 48’) and Yangshi (N 30° 21’, E 112° 55’) in Qianjiang city, Sancha town (N 30° 56’, E 114° 03’) and Dawu (N 31° 33’, E 114° 08’) in Xiaogan city.

**Fig 1:**
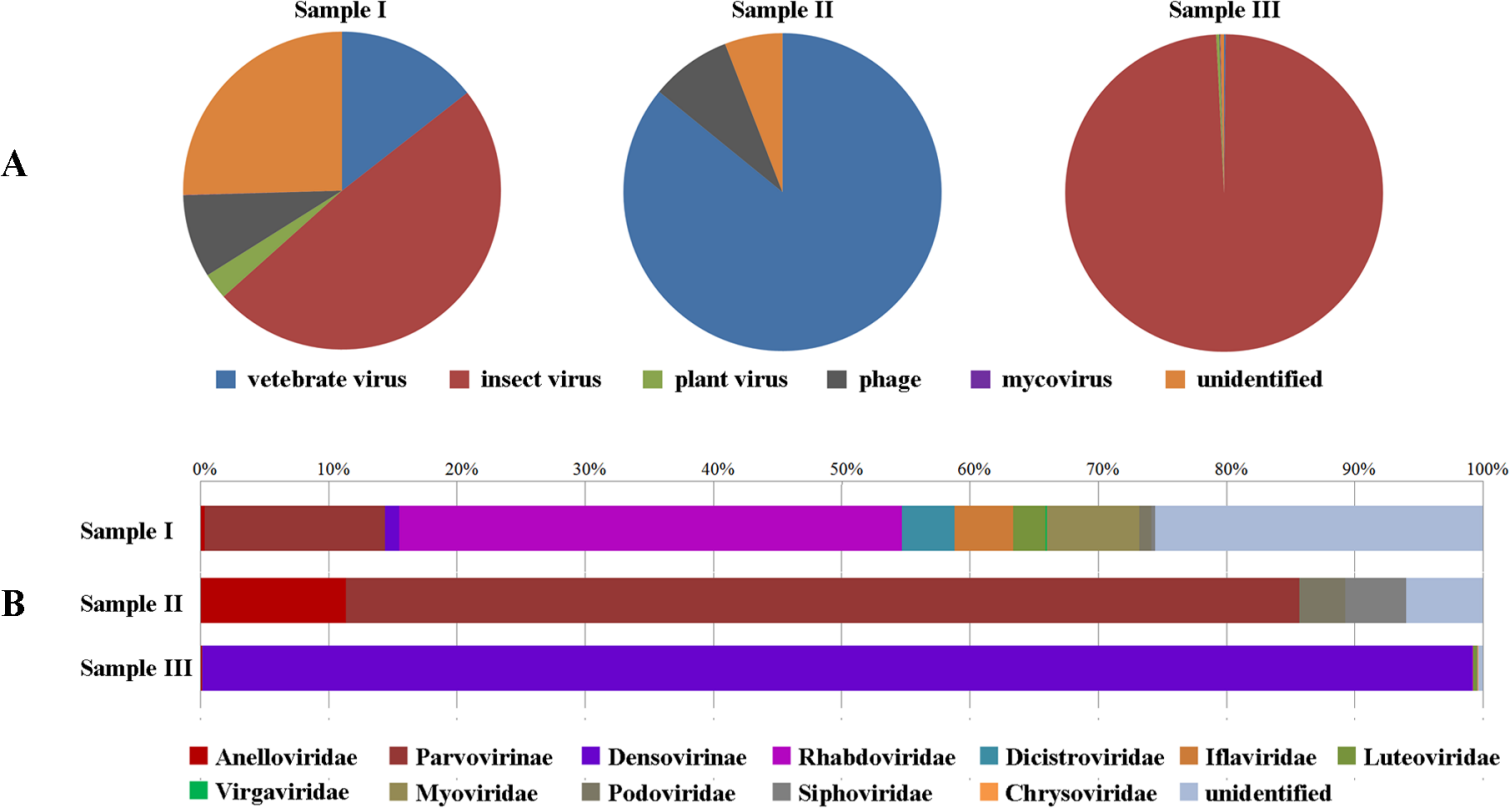
Taxonomic classification of viral sequences in three samples. (A) Viral sequences were classified by host type. Percentage of Mycovirus was too low which hardly can be seen from figure. (B) Viral sequences were classified on family level. The family with reads number less than 10 did not show in figure. Different host types and families were denoted with different colors.

The reads generated from the 454 High Throughput FLX short-gun sequencing were segregated into bins based upon barcode sequences and then the unique tags were trimmed from the ends. A total of 92,304 trimmed valid reads were generated after removing sequences shorter than 50 nt and the reads average length was 247 nt (Table 2). 39.2% viral sequences, identified by querying the viral database from NCBI running BLASTn and BLASTx at theE-value cutoff of 0.01, had high similarities to a variety of different viruses (Table 2), with 3.6% of vertebrate virus, 88% of insect virus, 0.8% of plant virus, 1.87% of bacteriophage and 0.03% of mycovirus (Fig 1A) among them. However, the abundance and diversity of viral sequences were different in three samples, with the largest viral reads number of Sample III and highest viral diversity in Sample I. Insect virus occupied the most in Samples III, but viral reads of Sample I scatteredly distributed in each virus type, which may attribute to the wide distribution of *C*. *tritaeniorhynchus*, the main vector of JEV in China. Though the gross reads of Sample II was less comparatively, the viral reads accounted for 21% and the majority were vertebrate viruses including Anelloviridae and Parvovirinae which also found in other samples.

**Table 2:** Basic Data of 454 FLX Sequencing.

Samples	Barcodes	No. of base	No. of reads	Ave. length (nt)	No. of viral reads	Percentage of viral reads
Sample I	A	12,416,751	51,123	242	7,298	14%
Sample II	B	190,921	823	231	170	21%
Sample III	C	10,786,900	40,359	267	28,717	71%
Total / Average		23,394,572	92,304	247	36,185	39%

All viral reads were classified on family level (Fig 1B). Among these viruses, some were biologically or mechanically transmitted by mosquitoes or specifically infected mosquitoes, the other were probably sucked sporadically by mosquito from host reservoirs. Additionally, considerable unidentified sequences highly likely belonged to some unexplored novel virus that dissimilative with known virus within mosquitoes, needing further investigation.

### Family Anelloviridae

Blast results showed that all three samples contained the viral sequences closely related to *torque tenosus virus1* (TTSuV1), Genus *Iotatorquevirus*, Family Anelloviridae. 8, 6 and 13 reads of each sample were assembled into one contigs with 745 nt, 339 nt, 484 nt separately. At nucleic acid level, contigs of Sample I and Sample III had high identity (nearly 99%) with isolated TTSuV1. In Sample II, contigs only showed 84% identity to known TTSuV1.

**Fig 2:**
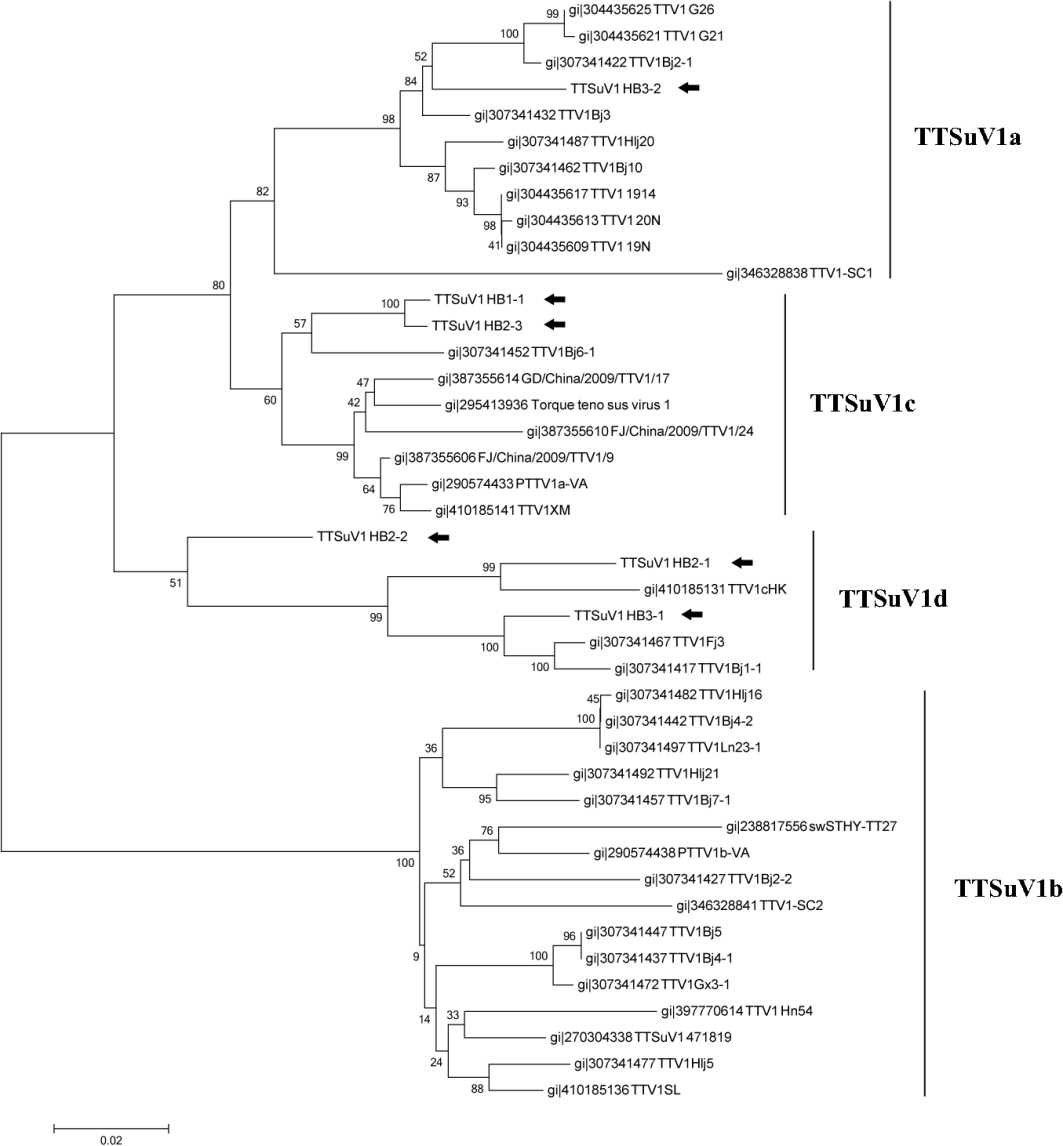
Neighbor joining phylogenetic tree of TTSuV1 based on 678 bp segment. 678 bp containeduntranslated region (UTR), the complete NS2 gene (ORF2) and 191 bp of the 5’end of capsid protein gene (ORF1). Six TTSuV1 sequences characterized in this study were marked with black arrow.

A pair of universal primers for TTSuV1 (Table 3) were used to amplify a 678 bp genomic fragment including the untranslated region (UTR), the complete non-structure protein 2 gene (ORF2) and 191 bp of the 5’end of capsid protein gene (ORF1) [19, 20] from the three samples. One sequence was identified in Sample I, three in Sample II and two in Sample III, which were named as TTSuV1 HB1, HB2-1/2/3 and HB3-1/2 respectively. These sequences have been deposited into GenBank (KR131713-KR131718). A neighbor-joining phylogenetic tree was constructed using the 6 amplified sequences and corresponding sequences of other 36 TTSuV1 stains in GenBank (Fig 2) and the identified TTSuV1 were closely related (> 90% identity) to known TTSuV1 isolated in China and subtyped into TTSuV1a, 1c and 1d [21]. Further analysis revealed that TTSuV1 HB1 and HB2-3 were in the same clade belonging to TTSuV1c with 96% identity to the strain TTV1Bj6-1. TTSuV1 HB2-1, HB2-2, and HB3-1 were classified into TTSuV1d with 93%, 90% and 97% similarity to strain TTV1cHK, Bj1-1 and Fj3 respectively. TTSuV1 HB3-2 had 97% identity to stain TTV1Bj3 in the subtype TTSuV1a.

**Table 3:** Primers used in this study.

Product names	Orientation	Sequence	Product size (bp)
I-PPV4 fragment1	F	ACAGTGTCTGAGAATCGCTCA	2022
	R	GTGACCGTGTTTCTGCATTGT	
I-PPV4 fragment2	F	GTGACCGTGTTTCTGCATTGT	1213
	R	GCTCATCACTTGTATCCTCTG	
I-PPV4 fragment3	F	TGACAATAACTCTGCTACACTG	1060
	R	GTGTTCGGTATCTGAGACCAT	
I-PPV5 fragment1	Ex-F^a^	TGCAATTGGAAAGAGTTGACTC	2385
	Ex-R^a^	ACCTGTGTAGCGATGTCCTG	
	In-F^b^	AGGTACAATAGGTGAAGCCTG	1955
	In-R^b^	CTTGATTGGTGATGTTCCTCAT	
I-PPV5 fragment2	Ex-F	AGTACCTGTGAGACCAGACAA	2596
	Ex-R	GGTTCCACCTCGGAGTATGT	
	In-F	ATCACTGCTGACTTCGATCAG	2386
	In-R	TTGCTTCCAGAACAGCTGGAT	
I-PPV6 fragment1	F	TATGTCTCAGACCTTCTGGAC	1667
	R	CAATCCTCTGGAGGAGTATCT	
I-PPV6 fragment2	Ex-F	TCACATCCAATACGGATATGTG	2266
	Ex-R	CAGCTCATACACAGGTTCTGT	
	In-F	ATTTCTGAAGATGAAGTCGTGG	1942
	In-R	ATGTCTCGCAGAAGATTCATCA	
I-PPV6 fragment3	F	TCTTCTGCGAGACATGACATC	1708
	R	CCTATACATGGTATGTCTGACA	
II-PPV4 fragment1	F	CATGGCTAAACAGTGTCTGAG	637
	R	GGATCTCTCCAACAGGAACTA	
II-PPV4 fragment2	F	GCTGGGCAAGGAATGTCAATT	286
	R	TCTCAACCGGAACTGCTGTAA	
II-PPV4 fragment3	F	TGATCAACTTGACGAAGCAGC	427
	R	CGCTGAGAGAACACATCTTCT	
II-PPV4 fragment4	F	AGTGAACCCATACATTGTGCC	410
	R	TGACCTGGCATCGAATATCCT	
III-DNV fragment1	Ex-F	GGAGAGCGAGATCAGTAATGA	2145
	Ex-R	GTGGCCGACTGATGAGTTTG	
	In-F	TGCATTTATTGTTGGGAGCATG	1904
	In-R	TGATCCATGTTGGCGTTCTCT	
III-DNV fragment2	Ex-F	TGTGTAGAGTGGATCGAATAC	1955
	Ex-R	GCTATATGGATCATCGGAGGT	
	In-F	GGAAGGCATAACAAATGCTGG	1797
	In-R	GGTGGACGTAGAAGGTGGAA	
I-BSRV fragment1	Ex-F	TCATTGTGTGGTTCTCTGGTG	3025
	Ex-R	CAGGTGCTGTTGTTCTGTCAA	
	In-F	GTCATATGGCATCGATATTGGA	2597
	In-R	TCTTGCTCCAAGACTGGTCG	
TTSuV1	F	CGGGTTCAGGAGGCTCAAT	678
	R	TCTACGTCCCCTCTACGG	

^a^Primer pairs for the first round of nested-PCR

^b^Primer pairs for the second round of nested-PCR

Although the distribution of TTSuV was investigated among 14 provinces of China [21], TTSuV1a, 1c and 1dwere firstly identified in mosquitoes from Hubei, suggesting the possible existence of the virus in pig population. The frequent variation of TTSuV had been noticed and it often co-infected with other viruses, especially the porcine circovirus, which posed potential threaten to pig herds [22].

### Subfamily Parvovirinae, Family Parvoviridae

**Fig 3:**
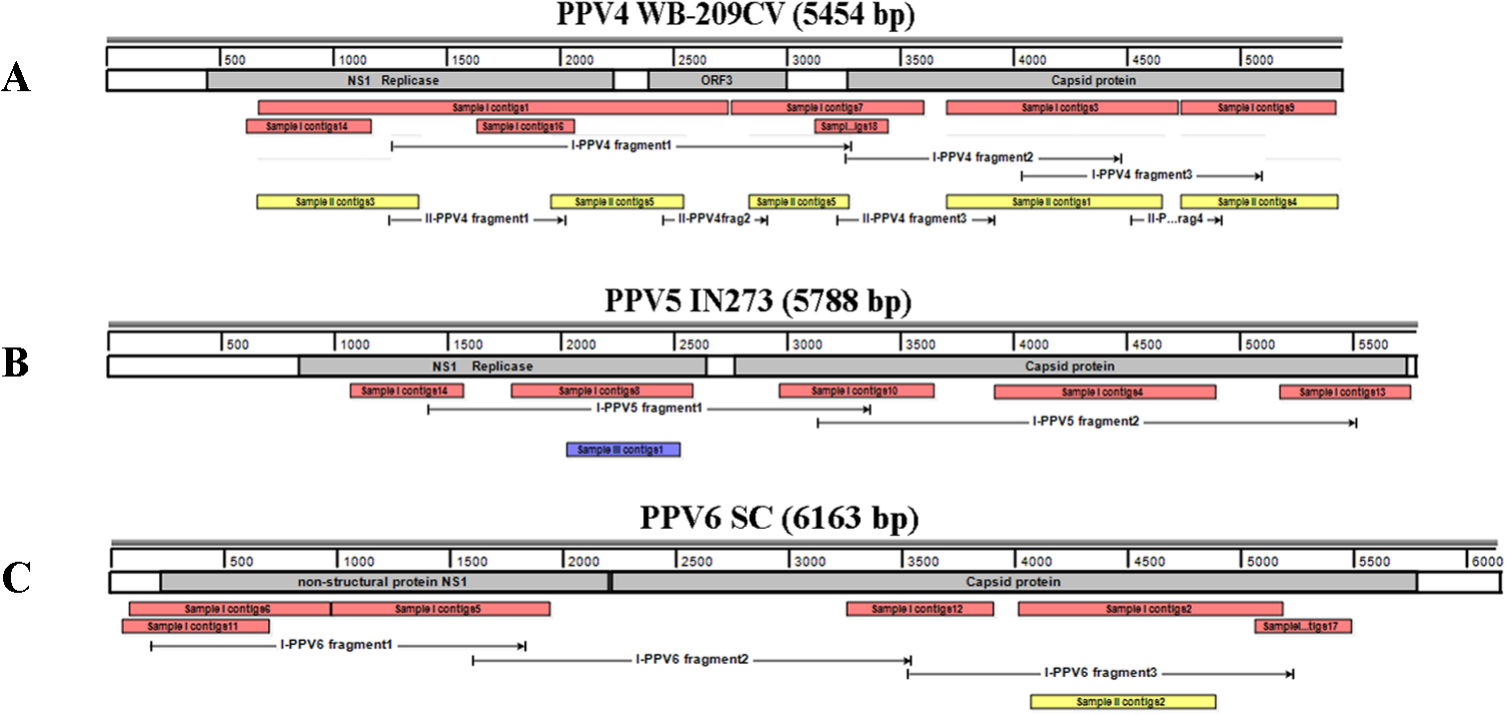
Alignment of PPV contigs in three samples. ORF of reference was labeled with grey strips. Contigs of three samples filled with red, yellow and purple respectively. Stroked arrow denoted the amplified fragments through PCR.

**Fig 4:**
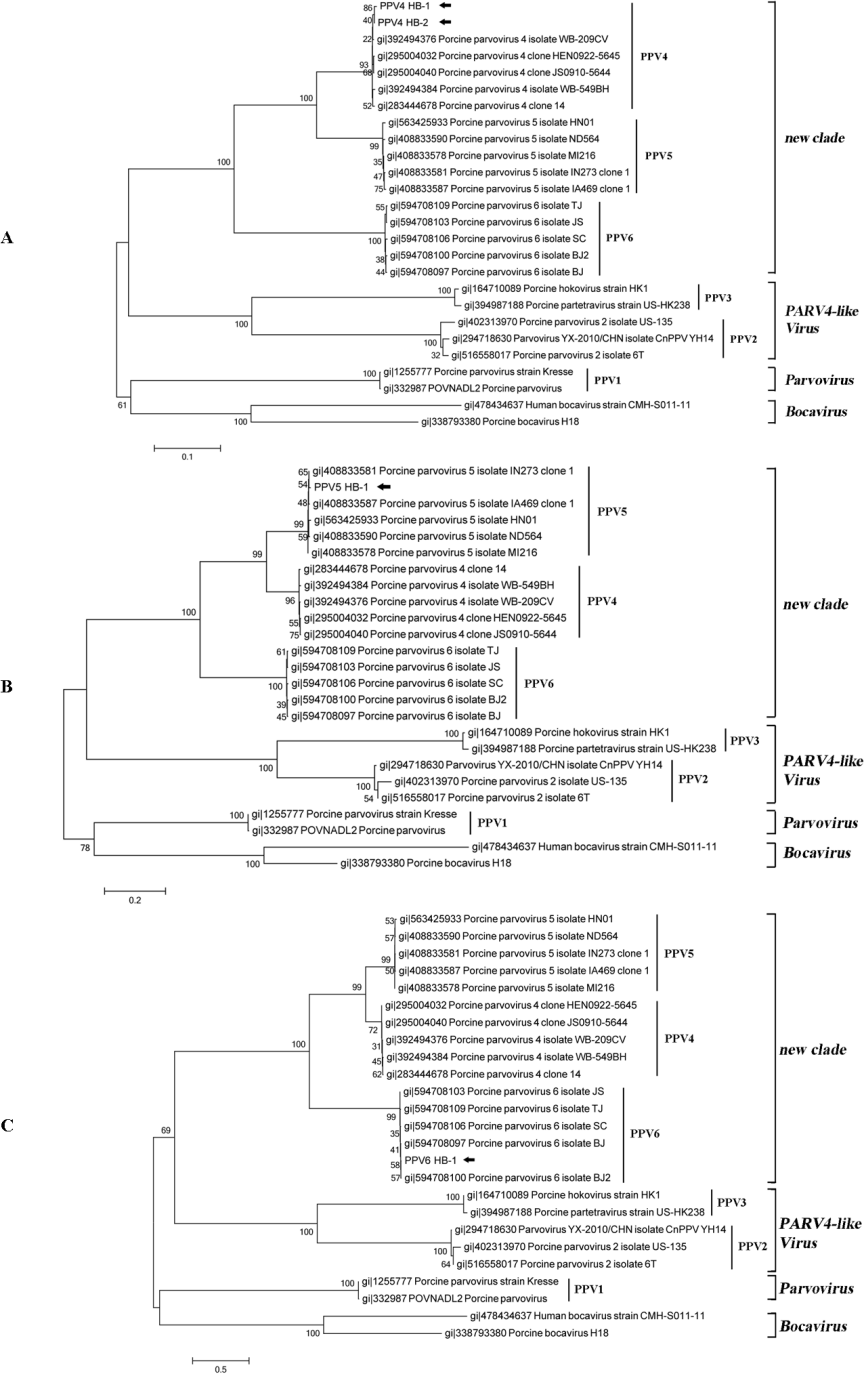
Neighbor joining phylogenetic tree of PPV based on nucleic acid of NS1. PPV sequences identified in this study were labeled with black arrows.

All three samples contained viral sequences belonging to Genus *Parvovirus*, subfamily Parvovirinae and the blast results suggested that these sequences matched most closely to porcine parvovirus (PPV). Sample I contained the majority of PPV sequences (991 reads), they assembled into 18 contigs (Table 4). Among them, seven contigs shared 99% nucleotide identity to PPV4, five were most closely related to PPV5 and six best matched with PPV6. 112 reads from sample II formed 6 contigs which were most closely to PPV4 (Table 4) and one contigs of Sample III comprised of 14 reads best matched with PPV5 (Table 4). PPV4 isolate WB-209CV (GenBank: JQ868714.1), PPV5 isolate IN273 clone 1 (GenBank: JX896319.1) and PPV6 isolate SC (GenBank: KF999684.1) were used as reference to map these contigs and the gaps were filled by PCR (Fig 3). The assembled contigs were aligned with other known PPVs and phylogenetic analysis results indicated all contigs have close relationship with the reference strains (Fig 4).

**Table 4:** Contigs Information of Porcine Parvovirus.

Sample	Contigs No.	No.of Assembled Reads	Length (nt)	Best match	Identity	E value
Sample I	1	298	2083	PPV4 WB-209CV	99%	0.0
Sample I	2	232	1323	PPV6 SC	99%	0.0
Sample I	3	54	1029	PPV4 WB-209CV	99%	0.0
Sample I	4	84	981	PPV5 IN273	99%	0.0
Sample I	5	14	972	PPV6 BJ	99%	0.0
Sample I	6	19	894	PPV6 BJ	100%	0.0
Sample I	7	16	854	PPV4 WB-209CV	99%	0.0
Sample I	8	165	806	PPV5 IN273	99%	0.0
Sample I	9	7	699	PPV4 WB-178SM	99%	0.0
Sample I	10	11	686	PPV5 IN273	99%	0.0
Sample I	11	8	655	PPV6 BJ	99%	0.0
Sample I	12	30	654	PPV6 BJ2	100%	0.0
Sample I	13	15	585	PPV5 IN273	99%	0.0
Sample I	14	14	540	PPV5 MI216	98%	0.0
Sample I	15	13	450	PPV6 JS	99%	0.0
Sample I	16	7	437	PPV4 WB-549HB	99%	0.0
Sample I	17	10	432	PPV6 SC	99%	0.0
Sample I	18	6	327	PPV4 WB-549HB	99%	2e-166
Sample II	1	32	1072	PPV4 WB-209CV	99%	0.0
Sample II	2	10	822	PPV6 SC	99%	0.0
Sample II	3	19	718	PPV4 WB-209CV	99%	0.0
Sample II	4	26	696	PPV4 F2-11SM	99%	0.0
Sample II	5	8	589	PPV4 WB-209CV	99%	0.0
Sample II	6	6	444	PPV4 WB-209CV	99%	0.0
Sample III	1	7	507	PPV5 IN273	99%	0.0

Porcine parvovirus (PPV) infection was always characterized by SMEDI (Stillbirth, Mummification, Embryonic Death and Infertility). The classical PPV (designated as PPV1) was first isolated in Germany and America in 1965 [23, 24] and has been proved wide distribution around the world especially in swine herds, causing economic loss for swine breeding industry. In recent years, a lot of novel PPVs had been identified using improving biology molecular technologies worldwide, but the classification and infection of these novel viruses were still unknown. The high mutation rate of capsid gene of PPV like RNA virus [25] and the evolution pressure of vaccine used currently might enhance the generation of novel PPVs. The increasing numbers of new PPVs discovered in different parts of the world indicated the endless evolution of PPV and suggested the vaccine produced from PPV strain decades years ago should be modified.

The presence of PPV and TTSuV1 in mosquitoes probably attributed to the contingent blood sucking of the mosquitoes from viremic pigs. Whether mosquito was an intermediate host to disseminate these porcine viruses or drew diseased blood coincidentally were needed to be verified in the subsequent study. Whatever, the mosquito control of pigpens was a crucial and effective step to keep pigs health.

### Subfamily Densovirinae, FamilyParvoviridae

**Fig 5:**
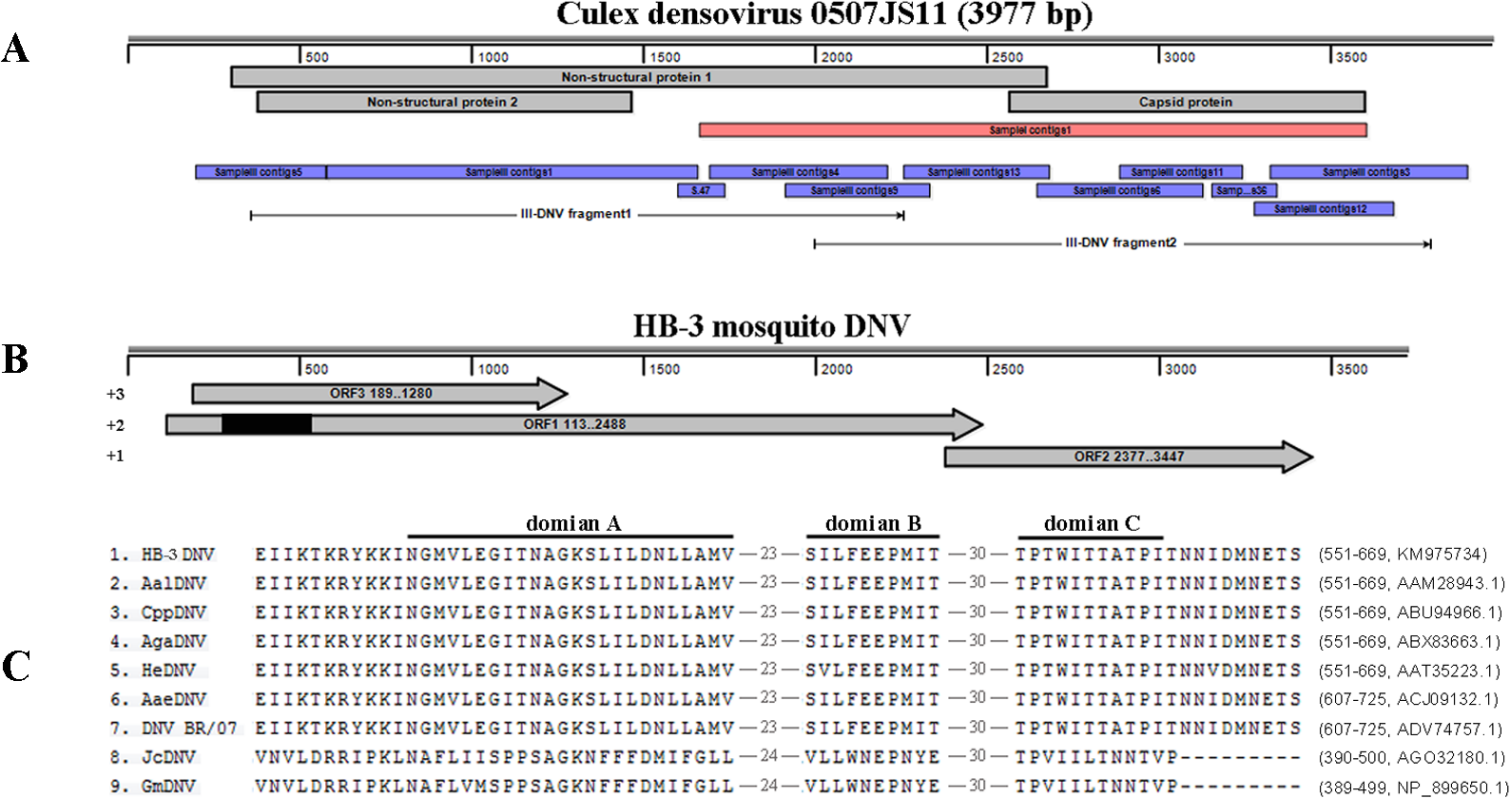
Amplification and sequence analysis of DNV in Sample III. (A) The mapping of DNV contigs in this study. It only presented the contigs used for assembly. ORF of reference was labeled with grey strips. Stroked arrow denoted the amplified fragments through PCR. (B) The ORF prediction of the sequenced novel DNV in Sample III. Black part (279 bp to 545 bp) of ORF1 was identified as Parvo_NS1 Superfamily. (C) 119 aa highly conserved region within NS1 of novel DNV like other DNVs. Domain A was the NTP-binding part. B and C were helicase domain.

Sample III contained the largest number of viral reads and majority of them had similarities with mosquito densovirus (DNV), genus *Brevidensovirus*, subfamily Densovirinae. More than 27,000 sequences were assembled into 58 contigs, of which the longest was 1084 nt. All contigs showed relative low identity (average identity was 85%) to known densovirus according to the BLASTn results. *Culex* densovirus 0507JS11 (GenBank: FJ805445.1) was used as a reference genome to align the 58 contigs which covered the whole reference genome (Fig 5). Four pairs of primers (Table 2) were designed referring to contigs nucleotide sequences and two nested PCRs were performed to amplify main genomic sequence. Two products, about 1791 bp and 1623 bp in length, were then cloned to vectors for sequencing, and a whole viral genome was assembled (KM975734), named as HB-3 mosquito DNV.

**Fig 6:**
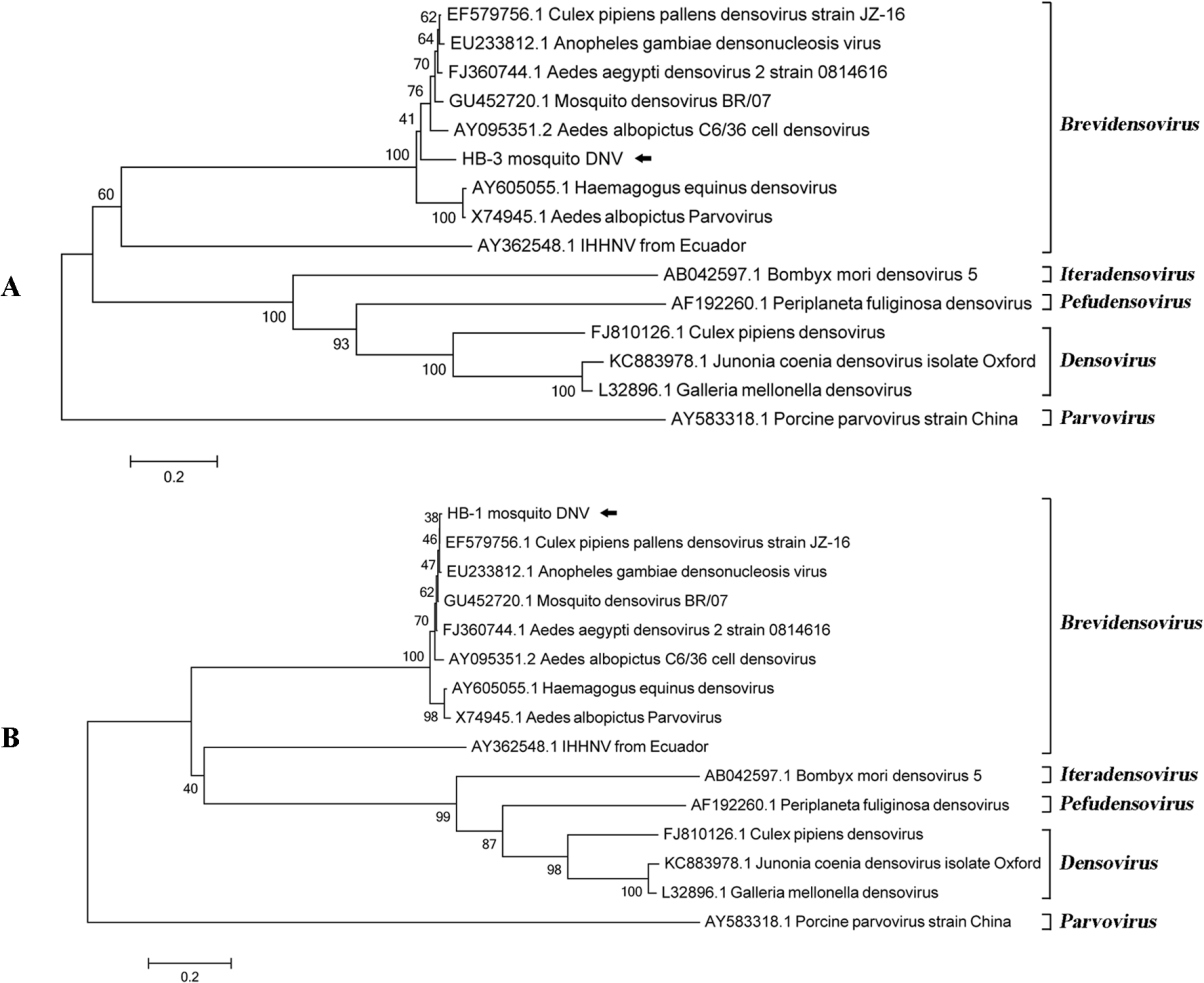
Phylogenetic analysis of DNV using neighbor joining method. (A) phylogenetic trees constructed based on aa sequence of putative ORF1 of HB-3 DNV and NS1 of other DNVs. (B) Partial nucleic acid sequence of Sample I contigs located in NS1 region was used for Phylogenetic analysis. DNVs identified in this study were marked with black arrows.

HB-3 mosquito DNV genome contained three ORFs through three different reading orders (Fig 5B). ORF3 encoded a protein of 363 amino acid (aa), having 73% similarity with non-structure protein 2 (NS2) of known DNVs. Translation product of the largest ORF1 with 791 aa showed 83% identity to NS1 of known DNVs. Besides, a highly conserved domain (279 bp to 545 bp) in ORF1, with the DNA helicase and ATPase activity, wasidentified as Parvo_NS1 Superfamily (http://www.ncbi.nlm.nih.gov/Structure/cdd/wrpsb.cgi?RID=Z5BKE39M013&mode=all). The right ORF2 encoded a product of 356 aa, sharing 82%aa identity to capsid protein of known DNVs. Multiple alignment of amino acid sequence revealed that NS1of HB-3 mosquito DNV had two highly conserved regions like other members of family Parvoviridae[26]. One from aa 316 to 375 (GNHLHILF-20-FILYCIRYGI) was the highly conserved replication initiator motif [27], another between aa 551 and 699 contained the NTP-binding sequence and helicase domain [27–29] (Fig 5C). Function of the most highly conserved protein NS2 whose aa sequence was distinctly different from others was still unknown. Capsid protein of HB-3 mosquito DNV involved a characterized highly conserved sequence (RGTKRKR, aa 14 to 20) in the glycine-rich region which also has been found in AaeDNV, AalDNV, CppDNV and HeDNV [30]. Amino acid sequence of NS1 was used for exploring the relationship between HB-3 mosquito DNV and other DNVs (Fig 6A), indicating that the novel DNV was apparent different from other mosquito DNVs. However, the characterization of HB-3 DNV should be performed for well understanding its biological property and its potential application.

Besides, 77 DNV reads in Sample I was assembled into one contigs with 1949 nt (Fig 5A). It showed <84% similarity at nucleotide level to known densoviruses and located in the latter half of DNV genome. The partial genesequence located in NS1 was used for phylogenetic analysis (Fig 6B), suggesting its close relationship with *Culex pipienspallens* densovirus.

Densovirinae includes three genus *Densovirus*, *Iteravirus and Brevidensovirus*. Among them, *Brevidensovirus* have been identified in or isolated from various mosquito cell line and wild caught mosquitoes, demonstrating the extensive distribution of mosquito densovirus [30, 31]. The viral type species of the genus was *Aedes aegypti* DNV (AaeDNV) isolated from mosquitolarvae [28], and some other DNVs were found in a chronically infected *A*. *albopictus* C6/36 cell line (AalDNV) [32], *C*. *pipiens* larvae (CpDNV) [33], and wild caught adult *C*. *pipiens pallens* (CppDNV) in China [30]. Besides one DNV isolated from shrimp (infectious hypodermal and hematopoietic necrosis virus, IHHNV) also showed high similarity to *Brevidensovirus* on genomic level [27].

The host ranges of mosquito DNV were narrow, but they can perpetuate through horizontal and vertical transmission in mosquitoes life cycle from infected adult to larvae [34]. Due to its high lethality to cell line and early instar larvae, as well as its persistent infection and transmissible viremiain late instar larvae and adult [31, 34] mosquito DNV could be used as viral agent for mosquito control.

### FamilyRhabdoviridae and Dicistroviridae

**Fig 7:**
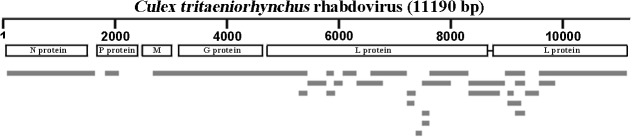
Alignment of CTRV contigs in Sample I. Grey strips were the contigs of CTRV in Sample I. Short line between two parts of L protein was the intron sequence.

The majority sequences in Sample I and several in Sample III matched to family Rhabdoviridae and all of them were most similar to *C*.*tritaeniorhynchus* rhabdovirus (CTRV), which has only been identified in Japan in 2011. Reads of Sample I formed 30 contigs with 98% identity to CTRV and covered the whole genome of CTRV (GenBank: AB604791.1) (Fig 7).

**Fig 8:**
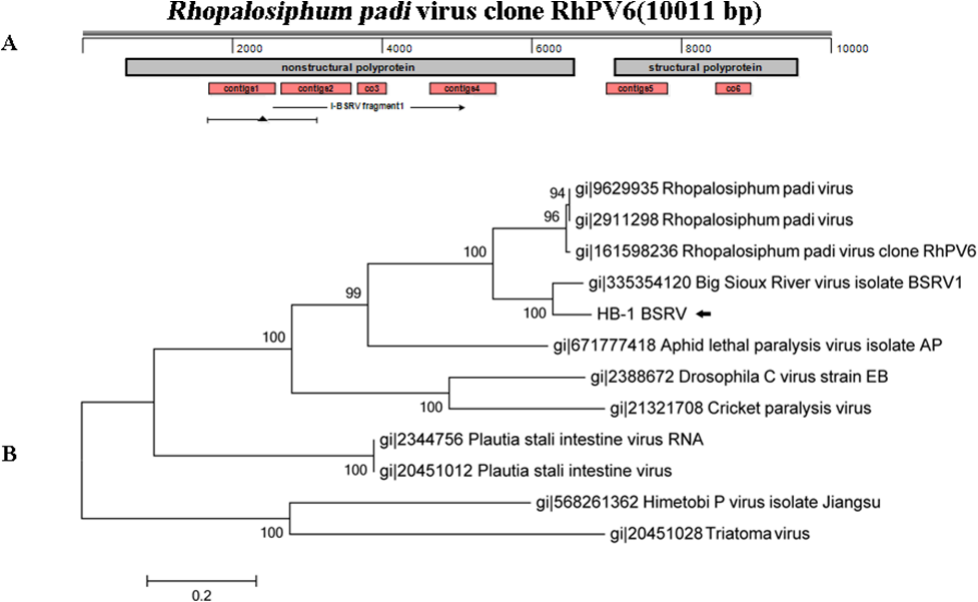
Alignment and phylogenetic analysis of contigs in Family Dicistroviridae of Sample I. (A) The mapping of contigs in Family Dicistroviridae of Sample I. ORF of reference was labeled with grey strips. Stroked arrowdenoted the amplified fragments through PCR. Triangle glyph with a line denoted the region used for phylogenetic analysis.(B) 1473 bp of the non-structure protein gene was used for phylogenetic analysis. BSRV detected in Sample I was labeled with black arrows.

Six contigs,assembledfrom the reads of Sample I, were most closely related to two species: *Rhopalosiphumpadi* virus (RhPV), Genus *Cripavirus* and Big Sioux River virus (BSRV), unclassified. On account that the BSRV had close relationship with RhPV [35] and only partial genomic sequences of BSRV could be found in GenBank, RhPV6 (GenBank: EU282007.1) was chosen to map the contigs (Fig 8A). Nested PCR was designed to amplify the gaps between the 4 contigs located in the gene of non-structure protein, denoted in Fig 8A. Futhermore, the 1473 bp sequence in nonstructural polyprotein gene of BSRV in GenBank was used for phylogenetic analysis with viruses identified in this study and other viruses in genus *Cripavirus* (Fig 8B). The result showed that newly identified sequences had closer relationship with BSRV in Family Dicistroviridae. HB-1 BSRV share 87% identity with BSRV1 and the E value was 0, which indicated likely to be a novel BSRV.

Rhabdoviruses had a wide range of hosts and had been identified in plants, vertebrate animals and insects [36]. However, no evidences showed their vertical transmission in plants and vertebrate animals, so insects as mechanical or biological vector played a part in their horizontal transmission [37, 38]. CTRV genome encoded 5 proteins like other rhabdoviruses, but its gene sequence of L protein, a RNA-dependent RNA polymerase, embraced an intron section which needed RNA splicing during transcription. This process was rare in negative single-strand RNA viruses and had never been found in other members of Family Rhabdoviridae [39]. In phylogenetic analysis, CTRV was closelyto genera *Vesiculovirus* and *Ephemerovirus* which mainly infectedvertebrate animals [39].

Partial genomic sequence of BSRV was only identified in western honey bees of USA in 2011. It most closely related to RhPV on the nucleic acid level, but shared only 78% and 69% aa identity with NS polyprotein and capsid protein of RhPV respectively, which met the taxonomic rank of a new species on the level of aa divergence [35]. The presence of BSRV in mosquitoes from China may indicated the wide existence of the virus, and its genome sequence, infectivity and transmission pathway need to be characterized in the following study.

## Materials and Methods

### Mosquito collection

Our mosquitoes were collected on private land and the owner of the land gave permission to conduct the study on this site. Sample collection was conducted with the help of Hubei CDC and no protected species were sampled, so no specific permissions were required for these locations/activities.

The adult mosquitoes were collected from the cowsheds and pigpens located in different residential area in Hubei province by using an electrical mosquito aspirator (Yalin, Zhejiang, China). The collected mosquitoes were transported to laboratory alive in cages and killed by freezing at -20°C for 30 min before classified into species with approximately 50 adult per pool. All mosquitoes were classed into three samples (I, II and III) for metagenomic analysis (Table 1).

### Nucleic acid extraction and library construction

Pools of mosquitoes were homogenized using TissueLyser II (Qiagen) and 3 mm tungsten carbide beads (Qiagen) in 1.5 ml sterile plastic tubes and 1 ml viral transport medium (VTM) containing Hank’s solution, 1% BSA, 15 μg/ml Amphotericin, 100 unit/ml Penicillin and 50 unit/ml Streptomycin. Samples were centrifuged at 13,000 × g for 30 min at 4°C, taking the supernatant for second centrifugation at the same conditions. Store the supernatant at -80°C until use.

120 μl supernatant was treated with Turbo DNA-free Kit (Ambion), Benzonase (Novagen) and RNase I (Fermentas) to digest unprotected nucleic acid. Nucleic acid packaged in viral capsids were extracted using QIAamp Viral RNA Mini Kit (Qiagen) without DNase digestion [11]. The extracts including both RNA and DNA viral sequences were used for reverse transcription with random primers containing short specific barcode sequences at 5’ end by M-MLV Reverse Transcriptase (Invitrogen), following by DNA extension reaction with Klenow fragment polymerase (Fermentas) and the same primers. Subsequently, PCR amplification was performed with Primer STAR HS (TaKaRa) and reverse random primers. PCR products were analyzed by 1% agarose gel electrophoresis. Then DNA between 500 to 1000 bp was cut and purified with gel extraction kit (Omega) and sequenced using Roche 454 GS FLX system. Three samples used different primers with specific barcode sequences (barcode A, B, C respectively) which could be recognized in the following analysis.

### Bioinformatics analysis and phylogenetic analysis

The reads of three samples were separated by barcode sequences and barcode sequences were trimmed. Trimmed reads that shorter than 50 nt were discardand then the remainder were compared against GenBank database using both BLASTn and BLASTx with E value set as 0.01. Sequences were divided according to the best match. Viral de novo DNA sequences within one species or genus were assembled using Newbler (Roche) [40] with the default parameters. Mapping of contigs used BLASTn. ORF Finder (http://www.ncbi.nlm.nih.gov/projects/gorf/) of NCBI was used for ORF prediction.

Multiple alignment of sequence was performed with ClustalW. All phylogenetic trees were performed with MEGA6 [41] using neighbor joining method and bootstrap set as 1000 replications.

### PCR amplification and Sequencing

Primers used in the study were in Table 4. Amplification of the gaps between contigs were conducted using Primer STAR HS (TaKaRa) and reaction condition was 98°C for 5 min, followed by 35 cycles of 98°C for 15 second, 57°C for 15 second, 72°C (extension time according to the length of product) and ended with a final extension of 10 min at 72°C. Reaction condition of 678 bp segment amplification of TTSuV1 was 94°C for 5 min, followed by 40 cycles of94°C for 30 s, 60°C for 45 sand 72°C for 3 min [20], with a final elongationstep at 72°C for 7 min. Then products were cloned to T-Vector pMD19 (Simple) (TaKaRa) for sequencing.
